# Responses of South Caspian coastal foraminifera to warming: spatial patterns and assemblage shifts

**DOI:** 10.1038/s41598-026-38207-1

**Published:** 2026-02-01

**Authors:** Hossein Bagheri, Mehrshad Taheri

**Affiliations:** Iranian National Institute for Oceanography and Atmospheric Sciences, Tehran, 1411813389 Iran

**Keywords:** Caspian sea, Temperature, Foraminifera, Sediments, Climate sciences, Ecology, Ecology, Environmental sciences, Ocean sciences

## Abstract

This study investigated the spatial distribution of coastal foraminifera at four stations, identifying seven distinct species representing five genera and three families, with *Ammonia beccarii caspica* as the dominant species. The Bandar Gaz station exhibited the highest density, species richness, evenness, and Shannon diversity. Consequently, Bandar Gaz was selected for a controlled microcosm experiment examining benthic foraminiferal community responses to increased water temperature (24 °C, 27 °C, and 30 °C) over 60 days. While the highest total density and evenness were observed at 30 °C, the total number of species and Margalef and Shannon indices did not significantly differ among treatments. Temperature changes significantly altered community structure through shifts in species dominance. *Ammonia* species displayed resilience and increased dominance with higher temperatures, replacing other species. *Elphidium advenum* density decreased significantly at 30 °C, while *Ammonia beccarri and Ammonia tepida* increased in dominance with rising temperatures. These findings highlight temperature-driven alterations in foraminiferal assemblages, with implications for coastal ecosystem monitoring in the context of climate change.

## Introduction

Global ocean warming, an undeniable outcome of climate change, poses a significant and increasing threat to marine ecosystems globally, including isolated aquatic environments^1,2^. The Caspian Sea, the world’s largest inland water body, exemplifies this vulnerability, displaying elevated temperatures that contribute to sea level fall and substantial ecological instability^3–5^. Notably, the southern Caspian is undergoing rapid warming^6,7^, resulting in shifts in species distribution, disrupted reproductive cycles, and altered migration patterns, all of which threaten the region’s unique biodiversity^8^. Rising water temperatures threaten the ecological integrity of benthic coastal animals like foraminifera, key sensitive organisms in marine ecosystems^9,10^. Climate models predict worsening conditions, with rising temperatures and diminishing rainfall compounding the Caspian Sea’s recession and further endangering its delicate ecosystems. These changes are projected to significantly impact benthic foraminifera, crucial indicators of marine health, affecting their distribution, abundance, biodiversity, and community structure.

Given the Caspian Sea’s importance as a biodiversity hotspot, the current lack of research on foraminifera, particularly along its southeastern coasts, represents a critical deficiency in our understanding and ability to safeguard this vulnerable ecosystem. Foraminifera serve as valuable proxies in both modern and paleo-environmental reconstructions, as evidenced by numerous studies^11–16^. While field observations provide extensive data, laboratory experiments elucidating foraminiferal responses to environmental stressors remain relatively limited^17–21^. These controlled studies, some simulating future global change scenarios and others exploring extreme conditions, are crucial for refining proxy calibrations and enhancing our understanding of foraminifera as environmental indicators^22^. Limited research suggests that foraminiferal response to environmental change is either rapid or delayed by several years^23^. Recent work has also explored temperature-induced shifts in entire benthic foraminiferal communities, documenting changes in species composition and diversity^24,25^.

In this study, our microcosm experiment investigates how temperature variations influence benthic foraminiferal communities, testing the hypothesis that warming significantly alters their structural composition and ecological functions. A controlled laboratory experiment was conducted to investigate the effects of elevated temperatures (24, 27, and 30 °C) on benthic foraminiferal assemblages from a coastal Caspian Sea community over a 60-day period. This research provides novel experimental data on the response of foraminiferal communities and species richness to temperature stress within a microcosm environment. The findings offer crucial insights into the potential impacts of climate change on Caspian Sea biodiversity, contributing to a better understanding of warming’s cascading ecological consequences. Furthermore, this laboratory culture-based study establishes a valuable reference point for both future field investigations and the refinement of paleotemperature reconstruction methodologies.

## Materials and methods

The Caspian Sea functions as a dynamic, non-tidal lake whose coastal and splash zones are shaped by powerful hydrodynamic processes, particularly wave action and rip currents. This ecologically sensitive region faces mounting pressures from multiple threats: anthropogenic disturbances (including physical habitat modification and pollution), natural stressors (such as coastal erosion and dramatic water level fluctuations), and the escalating impacts of climate change^5,26^. These compounding factors have significantly heightened the ecosystem’s vulnerability. Surface water temperatures exhibit extreme seasonal variability, fluctuating between 19.0 °C in winter months and rarely reaching to 30.0 °C during summer periods^7^, creating additional thermal stress on native species. Mazandaran and Golestan provinces are located in the south Caspian Sea, along the Iranian border. The southern Caspian coastal region supports over 3.5 million residents whose livelihoods depend primarily on agriculture, tourism, and fisheries.

**Fig. 1:**
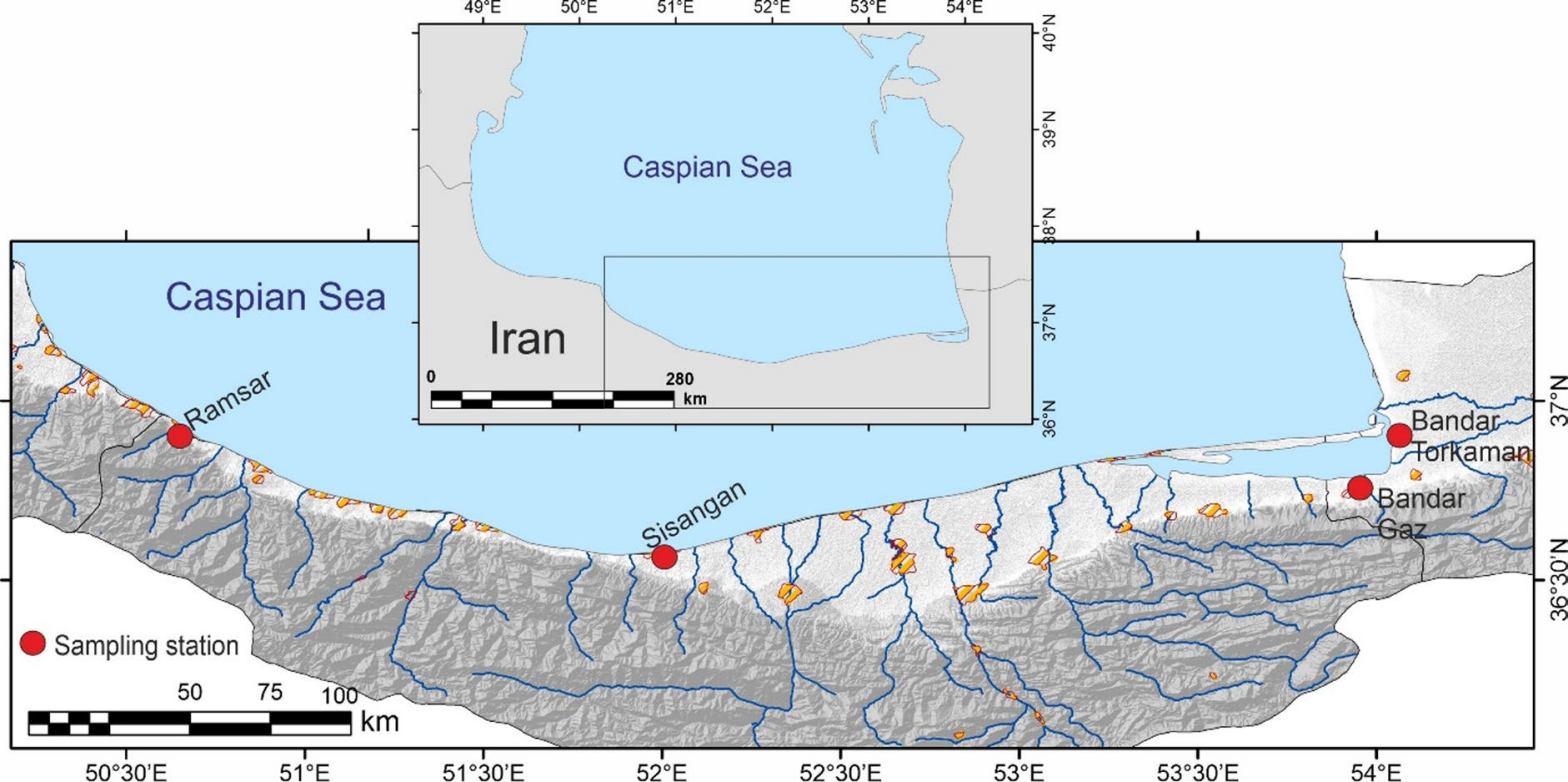
Map of the South Caspian Sea showing the four sediment sampling locations in Mazandaran and Golestan provinces, Iran. This map was created using QGIS software (version 3.28. ‘Firenze’, https://www.qgis.org).

In March 2023, we conducted sampling across four stations (Fig. 1) in Mazandaran and Golestan provinces (Bandar Torkaman, Bandar Gaz, Sisangan, and Ramsar) to assess the spatial distribution of benthic foraminifera in this low-biodiversity zone^27–29^. The map in Fig. 1 was created using QGIS geographic information system (version 3.28).

Sediment temperature, measured with a spirit thermometer (precision: 1 °C) in the surface layer (top 1 cm), ranged from 18 °C to 22 °C, while the annual average temperature is 23 °C. At each station, we collected three sediment samples using hand cores (5 cm diameter) from shallow waters (< 0.5 m depth), spaced 5 m apart along the shoreline. Each core penetrated 5 cm into the sediment to capture benthic communities. Samples were preserved in 4% buffered formaldehyde, stained with Rose Bengal^30^, and analyzed for total density, species richness, Shannon diversity, and evenness. Bandar Gaz station, showing the highest foraminiferal density and species richness, was selected for subsequent experimental work.

To investigate temperature effects on benthic foraminifera communities, we collected three replicate sediment samples from Bandar Gaz station using a hand corer (5 cm inner diameter). Surface sediments (0–5 cm depth) were subsampled and total organic matter (TOM) determined through loss-on-ignition (550 °C for 4 h) after drying to constant weight at 90 °C^31^. Sediment grain size distribution was analyzed using a HORIBA-LA950 Particle Size Analyzer (France & Japan). Resulting sediment fractions, categorized as gravel, sand, and silt+clay, were reported as percentage values and defined according to the Folk scale of particle size classification^32^. Furthermore, dissolved oxygen and pH of the overlying water at each core sampling location were measured in situ utilizing a HACH HQ40d portable multimeter (HACH, USA). Subsequently, 100 mL aliquots of the overlying water were collected from each core for laboratory analysis of nitrate, ammonium, phosphate, and silicate concentrations. These nutrient concentrations were determined spectrophotometrically using an Analytik-jena SPECORD-210 spectrophotometer, adhering to standard analytical protocols outlined in^33^. Sediment samples for biological analysis were collected along the coastal shoreline, maintaining a minimum distance of 5 m between sampling points. Twelve hand corers (internal diameter: 5 cm) were extracted to a depth of 10 cm within the splash zone (water edge ± 0.5 m) at Bandar Gaz, where the water temperature was 22 °C. Three cores were immediately processed in situ: these were sliced into 1 cm intervals down to a depth of 5 cm, and each slice was preserved in a buffered 4% formaldehyde solution. These field control (FC) samples were intended to preserve the characteristics of the foraminifera community at the time of collection^34,35^. The remaining nine cores were sealed at the bottom with rubber stoppers, covered with perforated plastic lids, and transported to the laboratory within 30 min. In the laboratory, the cores were randomly assigned to three separate 70-liter tanks. Each tank was filled with filtered seawater (38 μm filter) collected from the sampling site and maintained at 20 °C for five days^36^. During this period, the temperature within each tank was kept constant via a 200 W aquarium heater, and water homogenization was achieved by bubbling airstones, placed above the heaters, using an air pump^36^. The experiment was conducted using three tanks, each maintained at a distinct temperature: 24, 27, and 30 degrees Celsius. These controlled temperatures were achieved through the use of heaters. The effects of these varying temperatures were then observed and analyzed over a duration of 60 days. On the initial day of the experiment, and again after a 60-day incubation period prior to sediment slicing, dissolved oxygen and pH levels in the overlying water of each core were assessed using a portable multimeter (HACH HQ40d, USA). Additionally, 100 ml water samples were extracted from each core for the quantification of nitrate, ammonium, phosphate, and silicate concentrations, employing previously established methodologies. Following the 60-day incubation, foraminifera were picked up with a fine brush and stored on micropaleontological slides. Species were identified and counted using a stereomicroscope, in accordance with previously reported techniques^37,38^. Foraminifera were carefully extracted using a fine brush and mounted on micropaleontological slides. In addition, dead specimens in all samples were picked and counted in order to explore the percentage of living specimens in the foraminiferal assemblages.

### Statistical analyses

Statistical analyses were performed using PRIMER v6 with the PERMANOVA + add-on. At each sampling station and for each experimental treatment, species richness, Shannon diversity (H’, loge), and Pielou’s evenness (J) were calculated. Differences in univariate measures (total Foraminifera density, species richness, H, J) and multivariate community structure across stations and treatments were assessed using one-way PERMANOVA^34^. Significant PERMANOVA results were followed by Monte Carlo pairwise comparisons^39^. Bray-Curtis similarity was used to construct Non-metric Multidimensional Scaling (nMDS) plots, visualizing total and vertical Foraminifera community structure. Dominant species were identified as those comprising ≥ 5% of the assemblage at their minimum abundance temperature.

## Results

**Fig. 2:**
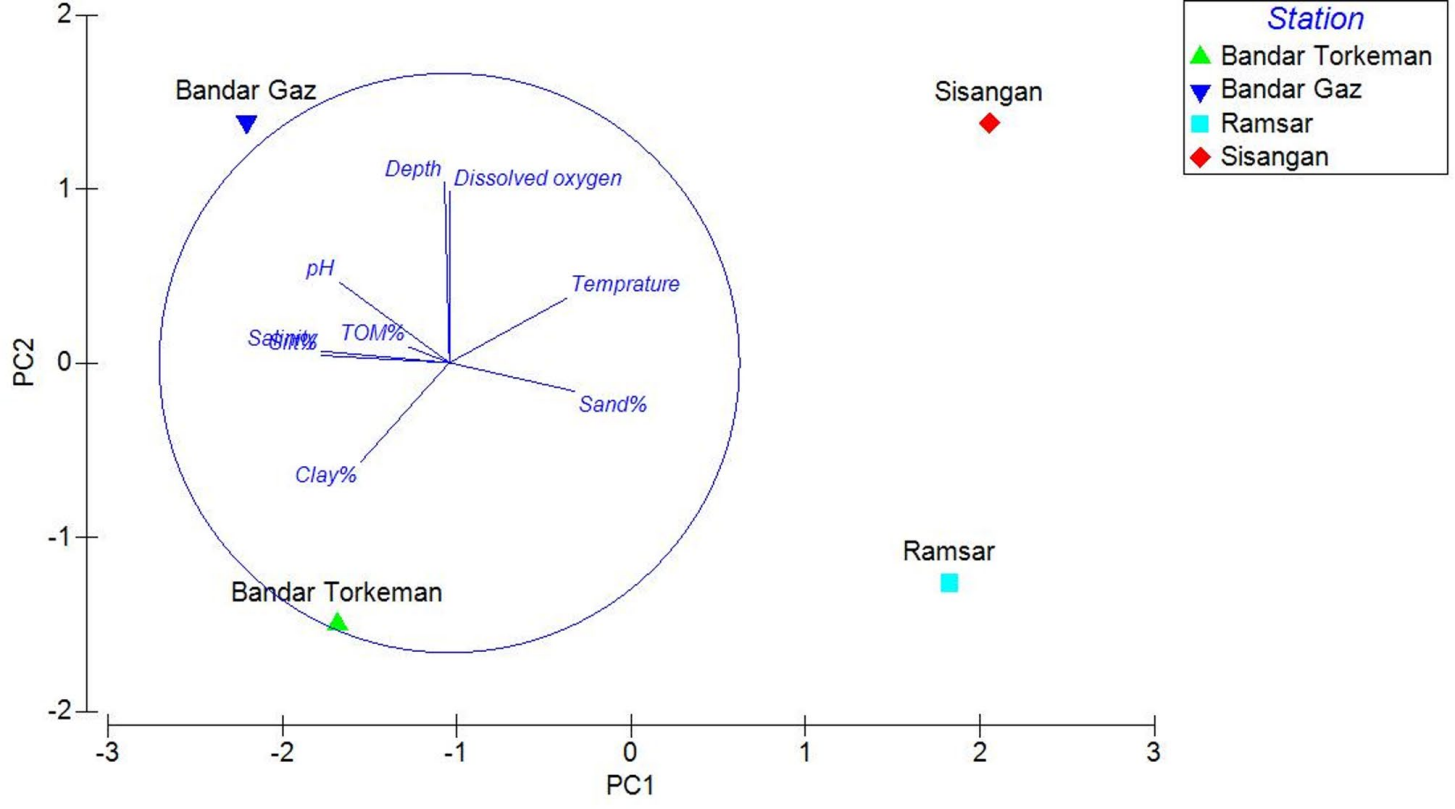
Principal Component Analysis of environmental parameters separated stations.

The presence of Foraminifera was confirmed across all sediment sampling locations. Subsequent analysis of collected samples identified seven distinct benthic species, belonging to five genera and three families, thereby demonstrating the diversity of these organisms within the studied environment. *Ammonia beccarii caspica*,* Ammonia tepida*,* Ammonia parkinsoniana*,* Elphidium advenum*,* Elphidium excavatum*,* Elphidium littorale caspicus*, and *Cornuspira* sp which were subsequently analyzed to characterize the foraminiferal community. The highest and lowest densities of foraminifera were observed at Bandar Gaz station (2497.5 individuals/0.1 m²) and Sisangan station (7.33 individuals/0.1 m²), respectively. *Ammonia beccarii caspica* was the dominant species in all sampling stations. *Ammonia tepida* and *Ammonia parkinsoniana* were not very abundance such as *Ammonia beccarii*. *Elphidium littorale caspicus* was observed in all stations and had the most abundance after *Ammonia beccarii* in the sampling area. *Elphidium excuavatum* and *Ammonia parkinsoniana* were rare in comparison with *Elphidium littorale*. *Cornuspira* sp only was observed in Bandar Torkaman. Principal Component Analysis of environmental parameters revealed that the first two principal components (PC1 and PC2) accounted for 84.90% of the total variance. Within PC1, silt and salinity exhibited notable negative loadings (−0.422), while temperature showed a significant positive loading (0.46). In PC2, dissolved oxygen (0.593) and depth (0.625) emerged as the most influential parameters in differentiating stations (Fig. 2).

Total density varied among stations (Pseudo-F = 5.31, *p* = 0.025). the lowest density was observed in Sisangan while there were not significant differences among the other stations. There was no difference in total number of species (Pseudo-F = 1.33, *p* = 0.80), evenness index (Pseudo-F = 0.77, *p* = 0.54) and shannon index (Pseudo-F = 0.84, *p* = 0.53) among stations. In total all three species from genus *Ammonia* did not show any significant differences among stations (all p values > 0.05) while there were dominant in total density. The density of *Elphidium littorale caspicus* varied among stations (Pseudo-F = 8.29, *p* = 0.01). the lowest density was observed in Sisangan while there were not significant differences among the other stations. Density of *Elphidium excavatum* also shows significant differences among stations (Pseudo-F = 5.88, *p* = 0.02). the lowest density was observed in Sisangan stations. The density of *Elphidium advenum* revealed a variation among stations (Pseudo-F = 56.25, *p* = 0.001). the highest density was observed at Bandar Torkeman station while the lowest was at Sisangan station (Table 1).

**Table 1:** Total foraminiferal density, number of species, evenness index, and Shannon index at all stations. Different capital letters above the columns indicate statistically significant results(A > B> C > D) of pairwise test(*p* < 0.05).

Total Density	Ramsar	Sisangan	Bandar Gaz	Bandar Torkaman
	4783 ± 1593.2^A^	1111 ± 190.47^B^	5560 ± 3632.38 ^A^	5160 ± 1659.7 ^A^
Number of Spicies	6.00 ± 00^A^	5.66 ± 0.57^A^	6.00 ± 00^A^	6.33 ± 0.57^A^
Eveness Index	0.76 ± 0.15^A^	0.62 ± 0.02^A^	0.62 ± 0.02^A^	0.70 ± 0.02^A^
Shannon Index	1.35 ± 0.27^A^	1.09 ± 0.27^A^	1.11 ± 0.04^A^	1.03 ± 0.08^A^
*Ammonia beccarii caspica*	2497.5 ± 1957.6^A^	726.67 ± 71.3^A^	2442.22 ± 1928.87^A^	1885 ± 86.63^A^
*Ammonia tepida*	352.92 ± 234.52^A^	101.61 ± 20.95^A^	332.78 ± 262.71^A^	280.83 ± 232.99^A^
*Ammonia parkinsoniana*	126.67 ± 7156^A^	60.00 ± 32.8^A^	97.78 ± 53.7^A^	93.33 ± 63.68^A^
*Elphidium littorale caspicus*	373.33 ± 211.36 ^A^	116.67 ± 63.42^B^	312.22 ± 195.89 ^A^	3.1.67 ± 217.52 ^A^
*Elphidium excavatum*	677.5 ± 495.32^A^	80.00 ± 24.49 ^B^	652.22 ± 562.3^AB^	430.00 ± 377.56^A^
*Elphidium advenum*	150 ± 115.54^B^	26.67 ± 17^D^	38.89 ± 13.34^C^	173.33 ± 147.95^A^

**Fig. 3:**
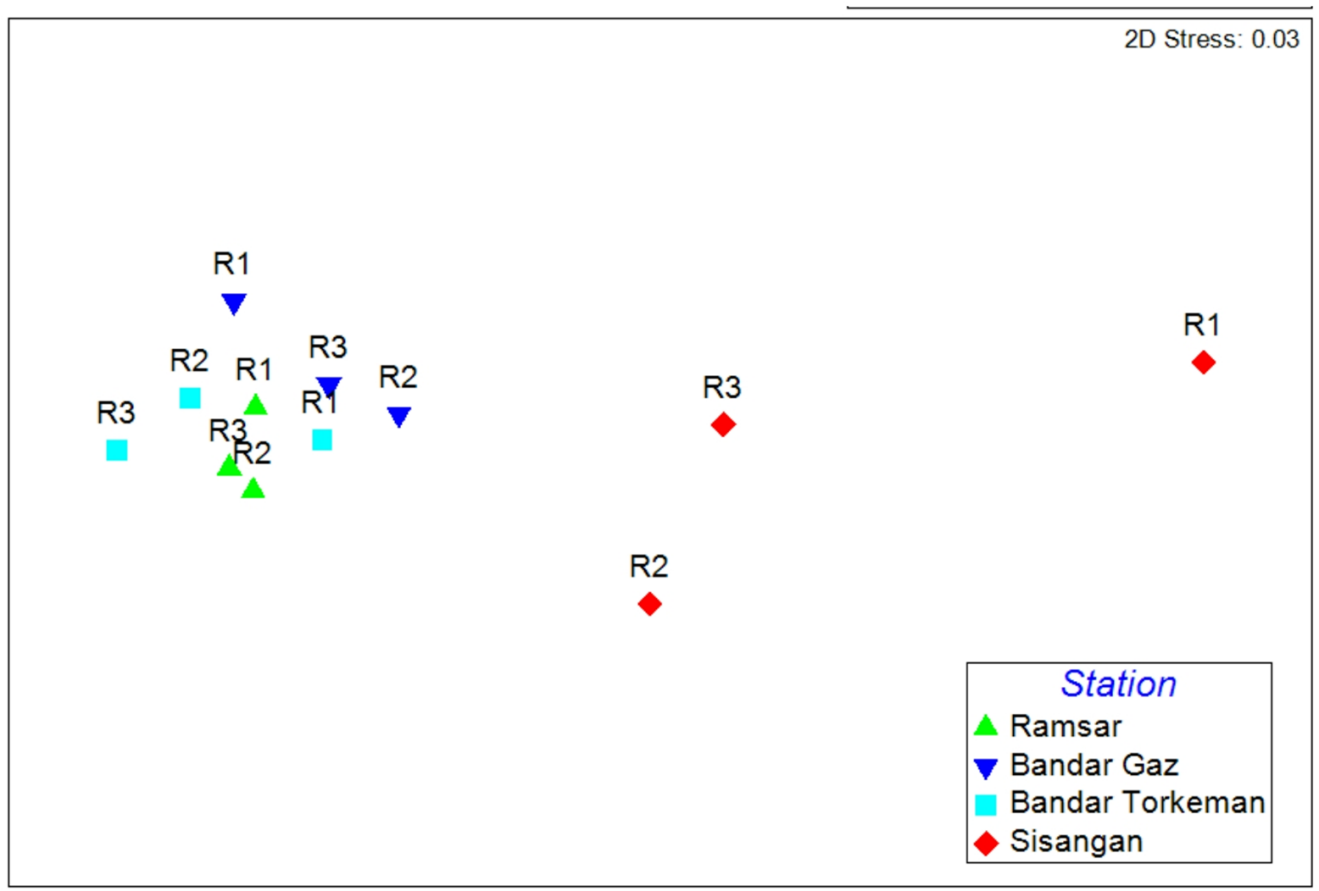
nMDS plot based on Bray–Curtis similarity on total foraminifera density data in different stations.

According to the nMDS plot based on Bray-Curtis similarity, there were significant differences in total foraminiferal density among stations (Fig. 3).

### Experimental study

Environmental parameters were carefully monitored throughout the experiment for T1 (24 °C), T2 (27 °C) and T3 for 30 °C (Table 2). Statistical analysis revealed no significant temporal variations in dissolved oxygen, pH, phosphate, or silicate concentrations across all treatments (PERMANOVA, *p* > 0.05). However, a marginally significant difference was detected in nitrate and ammonium levels between treatments (PERMANOVA, *p* < 0.05), with Treatment 1 exhibiting the lowest observed concentrations of these compounds.

**Table 2:** Mean environmental variables measured after 60 days for T1 (24 °C), T2 (27 °C), and T3 (30 °C). Of experiment. Different capital letters above the numbers indicate statistically significant results (A > B > C) Of pairwise test (*p* < 0.05).

Treatments	Do (mg/l)	pH	Nitrate (µg/l)	Ammonium (µg/l)	Phosphate (µg/l)	Silicate (µg/l)
T1, Firs	8.53 ± 0.21	8.08 ± 0.05	68.06 ± 3.00 ^B^	89.00 ± 19.31 ^B^	11.66 ± 2.08	176.00 ± 20.29
T1, Last	8.42 ± 0.21	8.22 ± 0.04	74.33 ± 3.05 ^B^	91.00 ± 16.09 ^B^	15.33 ± 0.04	169.05 ± 11.78
T2, First	8.52 ± 0.23	8.08 ± 0.04	92.66 ± 18.23 ^A^	97.33 ± 9.29 ^A^	13.66 ± 3.78	168.66 ± 19.55
T2, Last	8.56 ± 0.18	8.18 ± 0.06	98.66 ± 16.25 ^A^	101.66 ± 9.45 ^A^	15.56 ± 2.08	163.33 ± 20.10
T3, First	8.25 ± 0.11	8.11 ± 0.11	94.36 ± 9.50 ^A^	112.33 ± 4.93 ^A^	17.66 ± 3.51	178.33 ± 19.30
T3, Last	8.43 ± 0.20	8.23 ± 0.20	96.00 ± 5.29 ^A^	115.00 ± 4.58 ^A^	18.33 ± 1.52	170.33 ± 12.34

Overall, five foraminifera species were identified in the experimental study (Table 3). The species *Ammonia beccarii* was the most abundant groups in all treatments. However, Significant differences in density index were observed across treatments (Pseudo-F = 9.68, *p* = 0.005), with the highest density recorded at 30 °C and the lowest at 27 °C. The density of *Ammonia beccarii* also showed significant variation among treatments (Pseudo-F = 12.53, *p* = 0.032), peaking at 30 °C, while the other two treatments exhibited no discernible difference. *Ammonia tepida* density displayed significant differences across all treatments (Pseudo-F = 215.53, *p* = 0.006), with the highest density at 30 °C and the lowest in the initial treatment. The density of *Elphidium excavatum* significantly differed among treatments (Pseudo-F = 37.16, *p* = 0.003), with the highest density observed at 24 °C and the lowest at 30 °C (Table 3). Significant differences in *Elphidium advenum* densities were observed between the first and second, and first and third treatment groups (Pseudo-F = 31.553, *p* = 0.042), with overall significant differences between treatments. The highest density was recorded in the 24 °C treatment, while the lowest was observed at 30 °C. Similarly, *Elphidium incertum* exhibited significant differences between treatments (Pseudo-F = 7.21, *p* = 0.04), with treatment 3 displaying the highest abundance and treatment 1 the lowest (Table 3), and a lower abundance observed at 30 °C (Table 3).

**Fig. 4:**
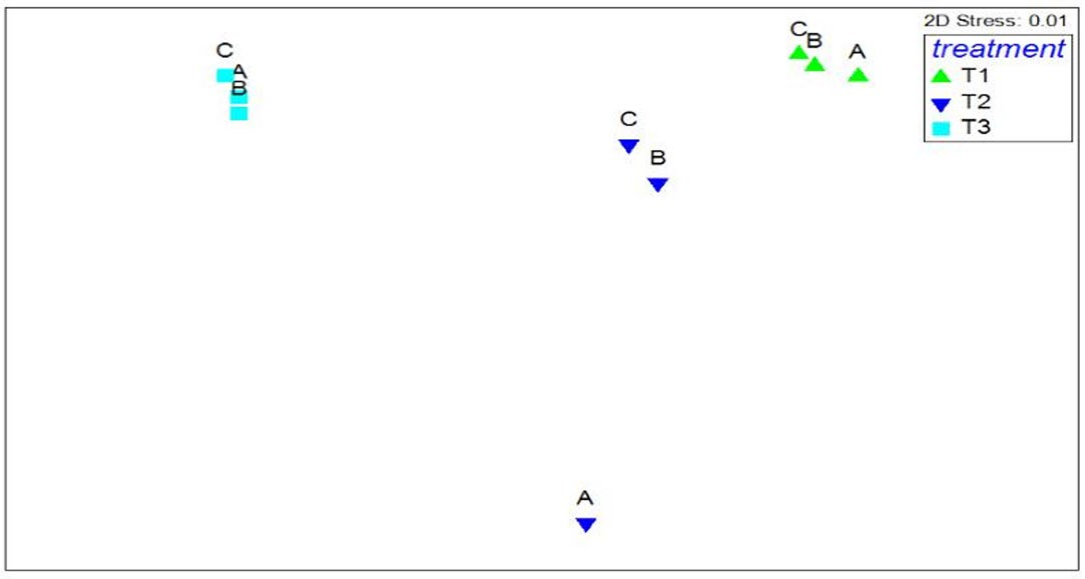
Multidimensional scaling (nMDS) plot illustrating the overall community structure of foraminifera.

The evenness index exhibited a statistically significant difference among treatments (Pseudo-F = 22.84, *p* = 0.012). While treatments 2 and 3 were not significantly different from each other, treatment 1 differed significantly from both. Shannon’s diversity index also revealed no significant difference between treatments 2 and 3 (Pseudo-F = 16.50 *p* = 0.036), but treatment 24 degrees differed significantly from treatments 27 and 30 degrees. No significant difference was observed among treatments with respect to Margalef’s index (Pseudo-F= 0.049, *p* = 0.663). Analysis of the community structure revealed a statistically significant difference between treatments and environmental controls (Pseudo-F = 5.52, *p* = 0.002). Multidimensional scaling (nMDS) plot clearly distinguishes the three temperature treatments (Fig. 4).

The analysis reveals temperature-driven shifts in species dominance. *Ammonia beccarii* exhibits resilience and prevalence across treatments, increasing in dominance with higher temperatures and replacing other species. While *Elphidium advenum* is also a dominant species, its density decreases significantly at 30 °C. Conversely, *Ammonia tepida*, initially less prevalent, increases its dominance as temperature rises, suggesting a preference for warmer conditions. These findings, detailed in Table 3, demonstrate the impact of temperature fluctuations on species composition.

**Table 3:** Mean density of various species at temperatures of 24, 27, And 30 °C. And biotic indices. (Different capital letters above the columns indicate statistically significant results(A > B> C > D) of pairwise test(*p* < 0.05).

Temperature	T1	T2	T3
*Ammonia beccarii*	743.33 ± 60.27^a^	713.33 ± 165.02 ^a^	1150 ± 43.58^b^
*Ammonia tepida*	13.33 ± 2.88 ^a^	23.33 ± 5.77 ^b^	153.3 ± 16.07^c^
*Elphidium excavatum*	135 ± 21.79 ^a^	40 ± 21.78 ^b^	6.66 ± 2.88^c^
*Elphidium advenum*	230 ± 10 ^a^	23.33 ± 20.2 ^b^	13.33 ± 5.77^b^
*Elphidium incertum*	1.66 ± 2.88 ^a^	6.66 ± 5.77^ab^	26.66 ± + 10.4 ^b^
Margalef index	0.56 ± 0.07^a^	0.58 ± 0.13^a^	0.63 ± 0.00^a^
Eveness Index	0.69 ± 0.04^a^	0.56 ± 0.03^ab^	0.53 ± 0.01^bc^
Shannon Index	1.15 ± 0.00^a^	0.92 ± 0.08^ab^	0.96 ± 0.02^bc^

## Discussion

This study offers valuable insights into the structure and distribution of foraminiferal communities in sediment samples from the south Caspian Sea. The experimentally derived temperature sensitivity data for dominant species suggest their potential application as paleotemperature proxies.The presence of seven identified species demonstrates notable biodiversity, emphasizing the ecological richness of these environments. *Ammonia beccarii caspica* was consistently the dominant species, while the relative abundance of other species varied significantly among the sampling sites. Such dominance has been observed in other studies, where *Ammonia* species are recognized for their resilience and adaptability to fluctuating environmental conditions^40,41^. In the present study, the presence of foraminifera across all sediment sampling locations indicates a widespread distribution of these organisms within the studied environment. Identification of seven distinct benthic species, belonging to five genera and three families, confirms notable diversity despite the relatively limited geographic scope of the study. The observed species composition, dominated by *Ammonia beccarii caspica*, suggests specific environmental preferences within the study area. Variation in foraminiferal density, ranging from high concentrations at Bandar Gaz to sparse populations at Sisangan, highlights the influence of localized environmental factors on their distribution. Also the relative scarcity of *Ammonia tepida* and *Ammonia parkinsoniana*, contrasted with the prevalence of *Elphidium littorale caspicus*, indicates niche differentiation or differential sensitivity to environmental factors. The restricted distribution of *Cornuspira* sp. to Bandar Torkaman warrants further investigation into localized habitat suitability. The highest density, number of species, and Shannon diversity were recorded at Bandar Gaz station. Additionally, significant differences were observed in the foraminifera community structure across the various stations. Consequently, Bandar Gaz station was selected for further experimentation.

Previous studies have demonstrated that foraminiferal communities can respond to environmental changes within just a few weeks^24,25^. Given this rapid responsiveness, our 60-day experimental period was sufficient to assess the effects of temperature on the benthic foraminiferal community in the south Caspian Sea. Species diversity and environmental correlates; principal component analysis highlighted key environmental drivers affecting foraminiferal distribution. The negative loadings of silt and salinity in PC1 suggest that finer sediments may be less favorable for certain foraminiferal species, while the positive association with temperature points to its crucial role in community structuring. Temperature is a well-documented factor influencing foraminiferal assemblages^42^, and our findings reinforce the concept that increasing temperatures can affect species dominance, as noted by the experimental results where *Ammonia beccarii* and *Ammonia tepida* exhibited increased densities at higher temperatures. These findings suggest that a complex interplay of sedimentological, thermal, and oxygen-related factors governs the distribution and abundance of foraminifera in this environment.

In addition the experiment meticulously monitored environmental parameters, revealing a stable environment with no significant temporal variations in dissolved oxygen, pH, phosphate, or silicate concentrations across treatments. Notably, nitrate and ammonium levels showed marginal differences between treatments, with Treatment 1 exhibiting the lowest concentrations. While *Ammonia beccarii* was the most abundant foraminifera species across all treatments, significant variations in density index, species densities, and community structure were observed among treatments, particularly driven by temperature. Specifically, higher temperatures (30 °C) promoted the dominance of *Ammonia beccarii* and *Ammonia tepida*, while depressing the density of *Elphidium advenum*. Conversely, *Elphidium excavatum* and *Elphidium advenum* thrived at lower temperatures (24 °C). These temperature-driven shifts in species dominance are further supported by differences in evenness and Shannon’s diversity indices between treatments, and are visually represented in the MDS plot demonstrating distinct community structures. Margalef’s index, however, remained consistent across treatments, suggesting that while species evenness and relative abundance shifted, overall species richness was unaffected by the experimental conditions.

In contrast, the fluctuating densities of species like *Elphidium advenum* across different temperature treatments highlight the sensitivity of certain species to environmental changes. This phenomenon aligns with findings by^8,43^, who documented shifts in benthic foraminifera populations in response to thermal stress. Finally, the observed trends suggest a general correlation between rising temperatures and increased density and biological indices in natural communities^44–46^. However, the impact of temperature is multifaceted and context-dependent. Community adaptation^47^, the magnitude of temperature change, and concurrent environmental factors such as pH, salinity^48^, and existing faunal composition all modulate the observed response. Further research is needed to disentangle the complex interactions driving these shifts.

## Conclusions

By integrating field observations with controlled warming experiments, this study demonstrates the temperature sensitivity of Caspian Sea benthic foraminifera. While natural assemblages are influenced by multiple environmental factors, our experiments isolate temperature as a key driver of change, leading to a systematic shift in species dominance. The increased relative abundance of *Ammonia beccarii* and *Ammonia tepida* at the expense of *Elphidium* sp. under warmer conditions suggests a directional change in community composition under future warming. Consequently, continued climate warming is projected to homogenize these ecosystems toward stress-tolerant taxa, with potential implications for biodiversity and benthic food-web structure. These findings establish a critical baseline for monitoring ecological change in the Caspian Sea and highlight the need for further research to clarify the complex interplay of environmental factors shaping foraminiferal communities in this vulnerable ecosystem.

## Data Availability

The datasets during and/or analyzed during the current study available from the corresponding author on reasonable request.
